# Supplementary material for: Synthesis, Characterization, and Reactivity Studies of New Cyclam-Based Y(III) Complexes
Source: Molecules. 2023 Dec 7;28(24):7998. doi: 10.3390/molecules28247998 (PMC10745738; doi:10.3390/molecules28247998)
Supplement: Supplementary file 1 [file molecules-28-07998-s001.zip › molecules-2763537-supplementary.pdf]

Synthesis, characterization, and reactivity studies of new cyclam-based Y(III) complexes

SUPPORTING INFORMATION

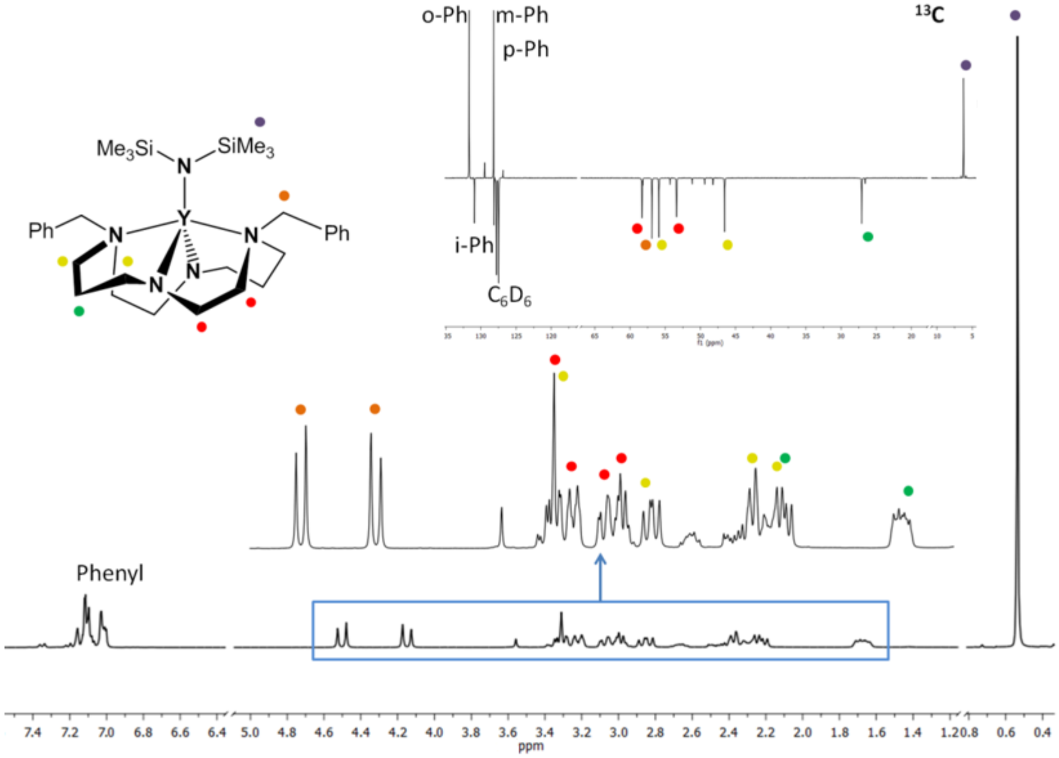

Figure S1. ^{1}H NMR and $^{13}C\{^{1}H\}$ APT spectra of **2** in C_6D_6. The corresponding protons and carbons are identified with coloured dots.

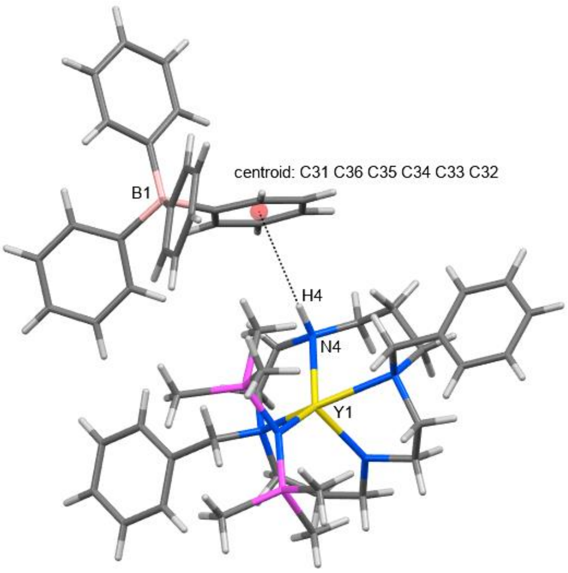

Figure S2. N-H···π interactions in [(HBn$_2$Cyclam)Y(N(SiMe$_3$)$_2$)][BPh$_4$], **3**.

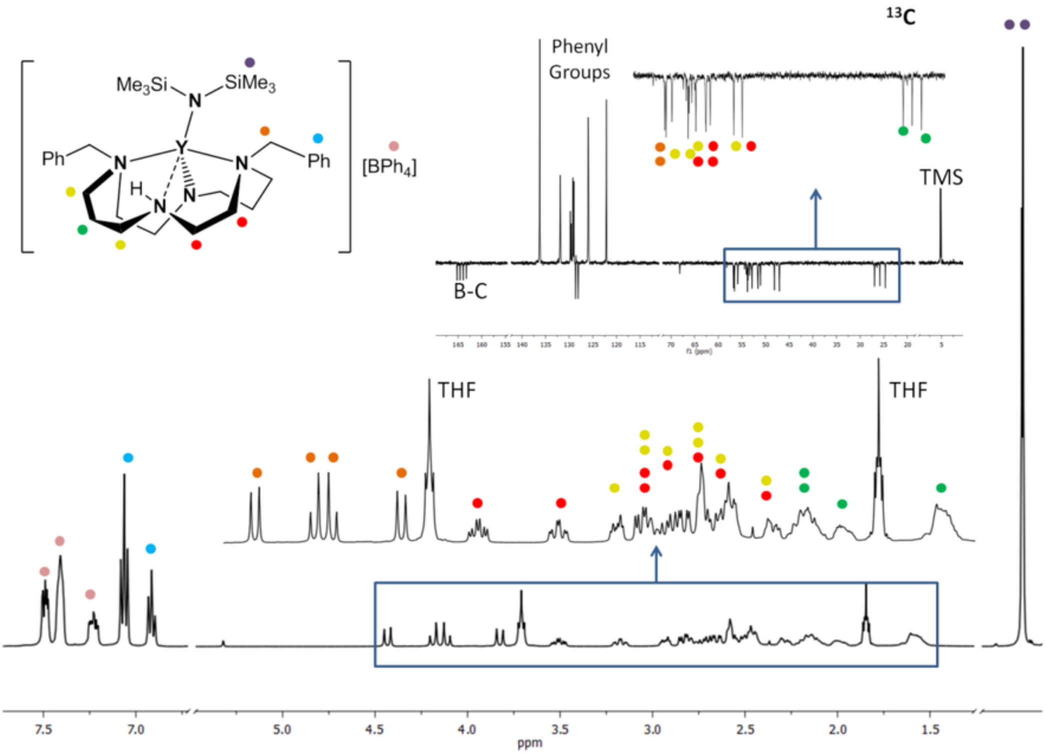

Figure S3. ^{1}H NMR and ^{13}C{^{1}H} APT spectra of **3** in CD_2Cl_2. The corresponding protons and carbons are identified with coloured dots.

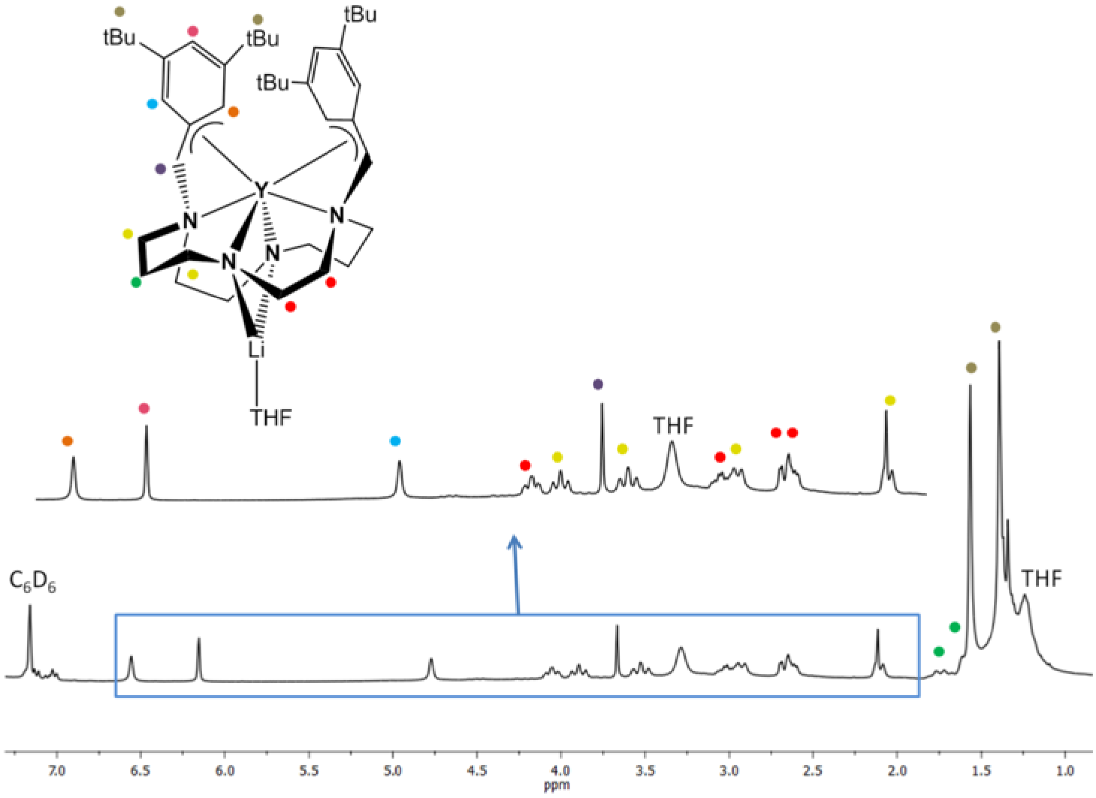

Figure S4. ^{1}H NMR spectrum of **6** in C_6D_6. The corresponding protons are identified with coloured dots.

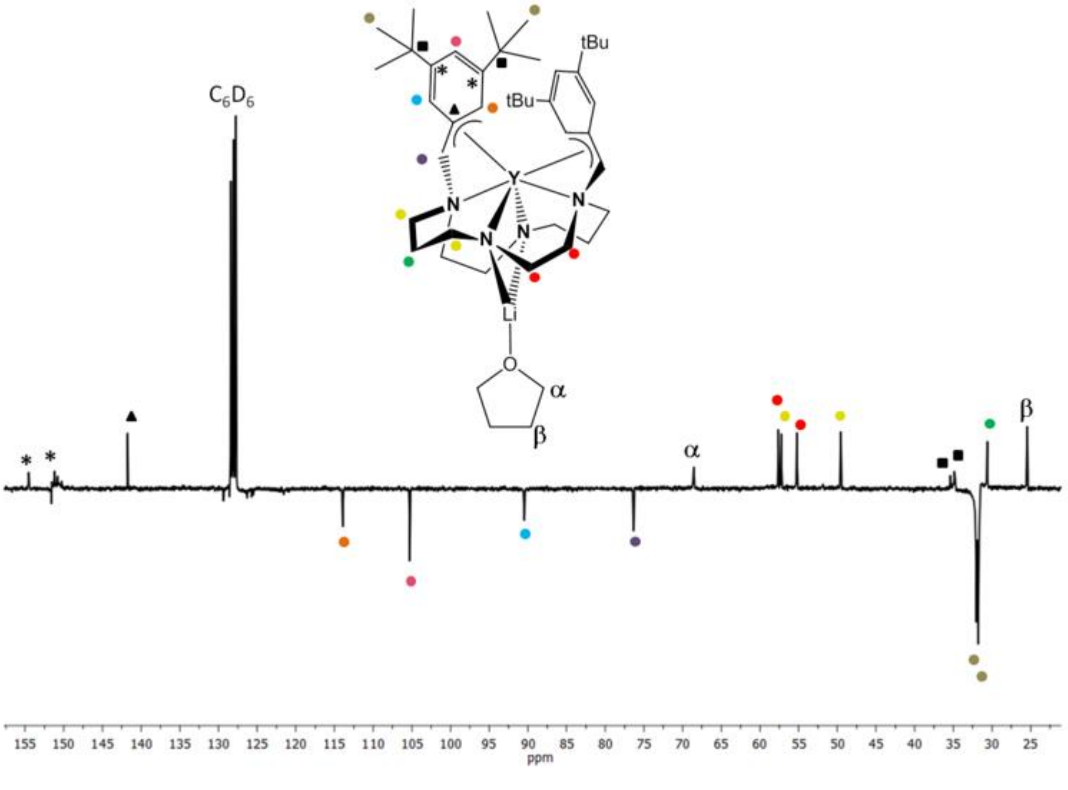

Figure S5. $^{13}C\{^{1}H\}$ APT NMR spectrum of **6** in C_6D_6. The corresponding carbons are identified with coloured dots, asterisks (*), squares (■) and triangles (▲).

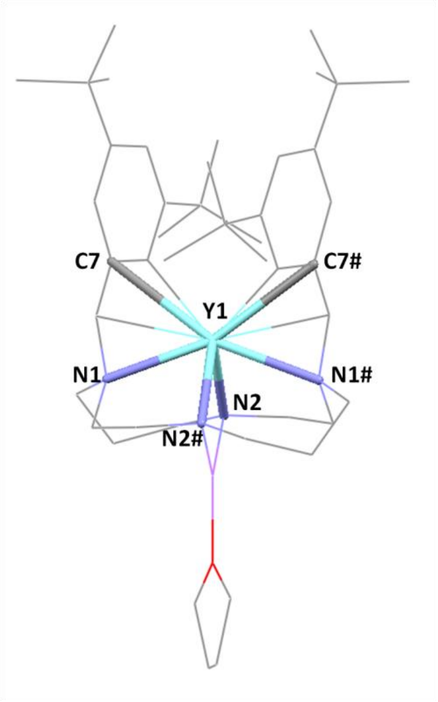

Figure S6. Illustration of the molecular structure of [Y{(η[3]-$^{3,5\text{-}tBu2}$Bn)$_2$Cyclam}Li(THF)], **6**, highlighting the coordination geometry around the metal center.

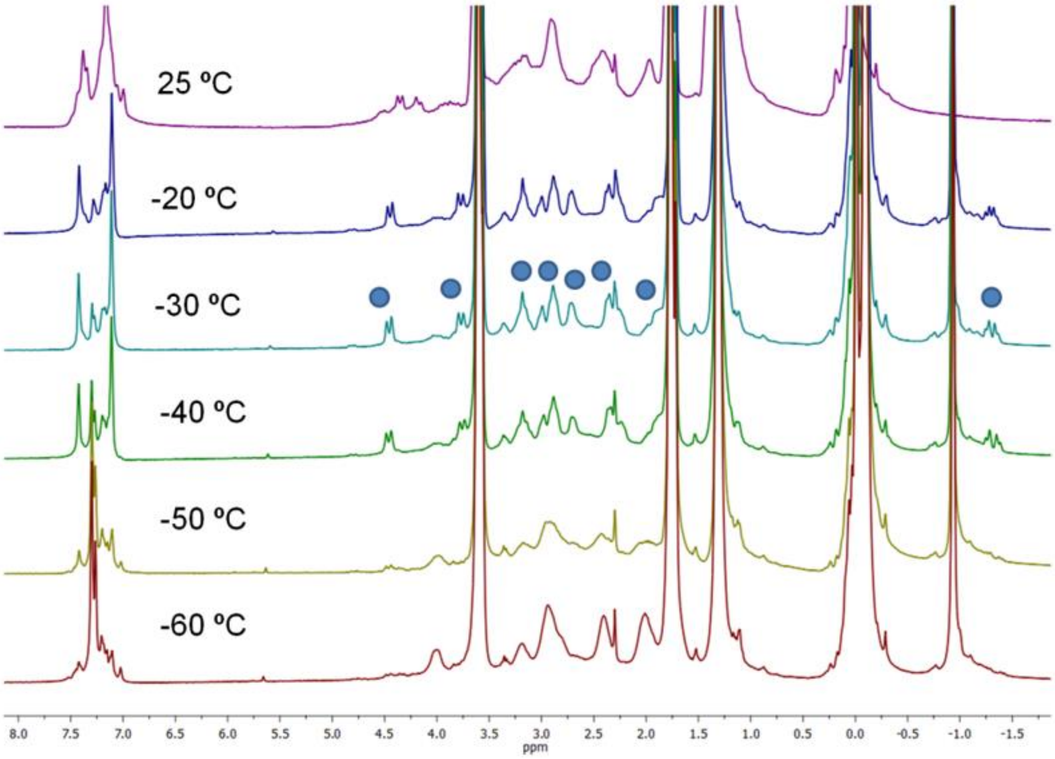

Figure S7. Variable temperature ^{1}H NMR spectra of the reaction between $Y(CH_2SiMe_3)_3(THF)_2$ and **5** in THF-d_8. The blue dots identify a new C_2-symmetric species at –30 °C.

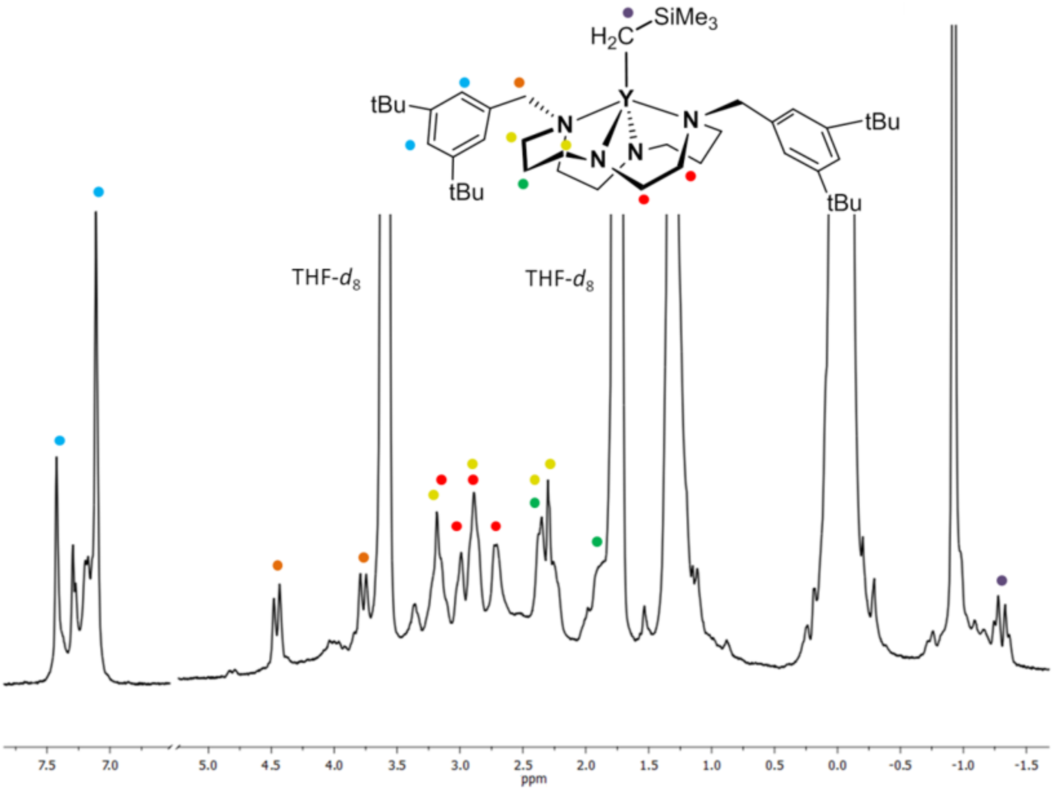

Figure S8. ^{1}H NMR spectrum of [($^{3,5\text{-}tBu2}Bn_2Cyclam$)Y($CH_2SiMe_3$)], **7**, at –30 °C in THF-*d*$_8$. The corresponding protons are identified by the coloured dots.

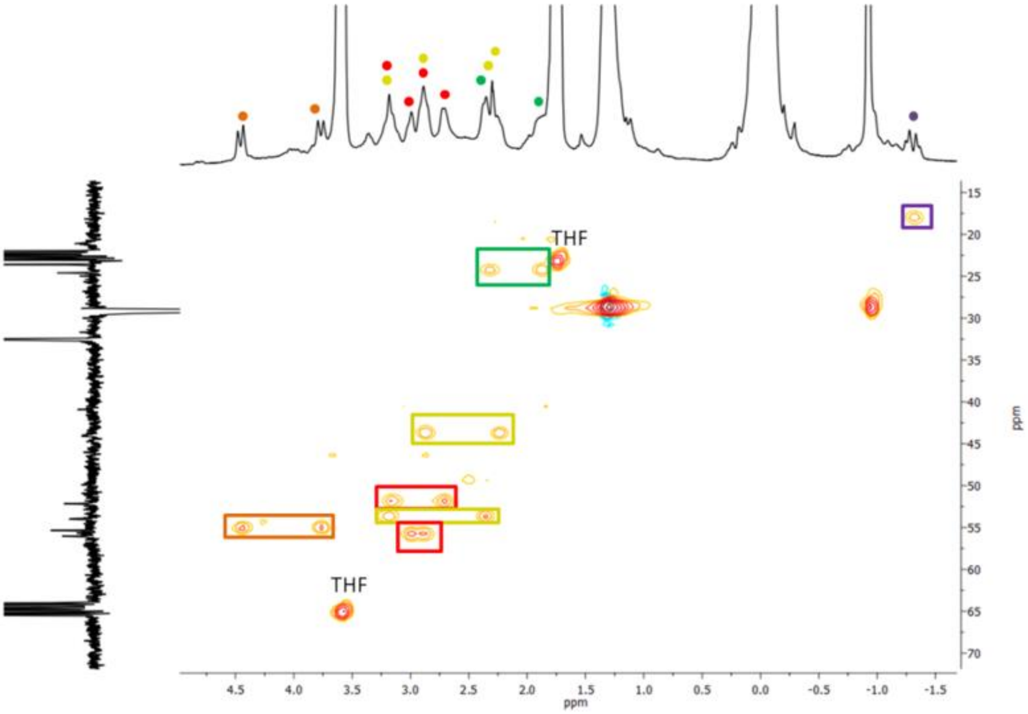

Figure S9. ^{1}H-$^{13}C\{^{1}H\}$ HSQC NMR spectrum of [($^{3,5\text{-}tBu2}Bn_2Cyclam$)Y($CH_2SiMe_3$)], **7**, at –30 °C in THF-*d*$_8$. The corresponding protons are identified by the coloured dots.

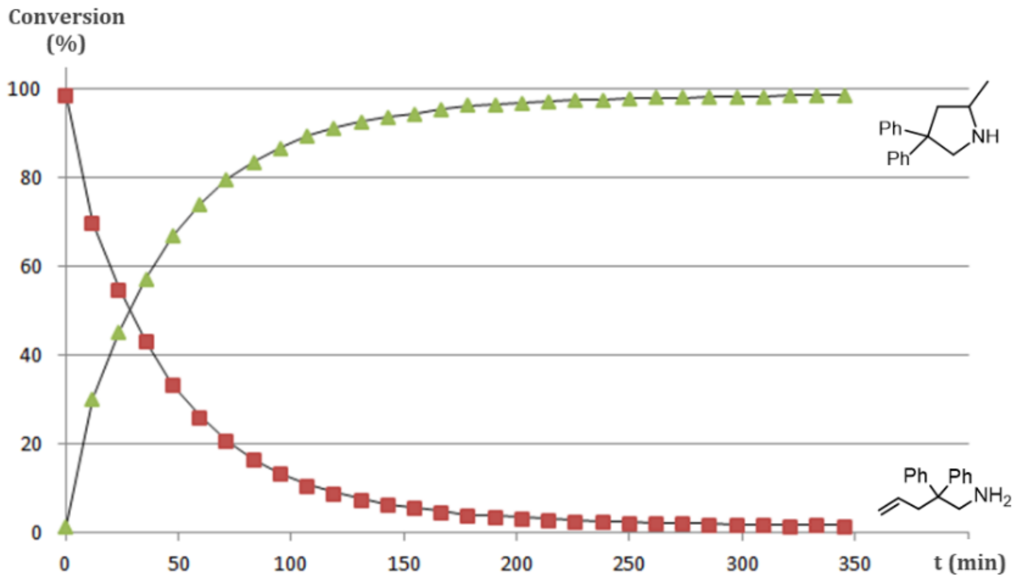

Figure S10. Cyclization kinetics of 2,2-diphenyl-pent-4-enylamine catalysed by **6** at room temperature.

Table S1. Crystal data and structure refinement for **2**-**4** and **6**.

	**2**	**3**	**4**	**6**
Formula	$C_{30}H_{52}N_5Si_2Y$	$C_{54}H_{73}BN_5Si_2Y$	$C_{40}H_{65}ClLiN_4O_4Y$	$C_{44}H_{72}LiN_4OY$
F_w	627.86	948.07	797.26	768.91
Crystal system	Triclinic	Triclinic	Triclinic	Monoclinic
Space group	P-1	P-1	P-1	I2/c
a, Å	9.829(2)	11.878(1)	9.5832(8)	17.5940(9)
b, Å	10.537(2)	16.334(2)	12.368(1)	16.562(1)
c, Å	17.630(2)	19.226(2)	18.243(2)	18.698(2)
α, °	100.138(6)	104.917(5)	78.965(5)	90
β, °	98.842(7)	107.311(6)	81.416(6)	108.378(3)
γ, °	110.746(6)	105.236(5)	73.368(5)	90
V, Å^3	1633.9(6)	3199.8(6)	2023.2(3)	5170.6(7)
Z	2	2	2	4
D_c, g.cm^{-1}	1.276	0.984	1.309	0.988
μ(Mo Kα), mm^{-1}	1.885	0.981	1.551	1.158
F(000)	668	1008	848	1656
Crystal size (mm)	0.06 x 0.20 x 0.20	0.10 x 0.20 x 0.20	0.20 x 0.20 x 0.20	0.30 x 0.40 x 0.60
θ range (°)	2.174 – 25.489	1.191 – 25.485	1.926 – 25.363	1.682 – 25.839
Limiting indices	$-11 \le h \le 11$, $-12 \le k \le 12$, $-21 \le l \le 21$	$-14 \le h \le 11$, $-19 \le k \le 19$, $-20 \le l \le 23$	$-11 \le h \le 11$, $-14 \le k \le 14$, $-20 \le l \le 21$	$-21 \le h \le 21$, $-20 \le k \le 20$, $-22 \le l \le 22$
Refl. collected/unique [R_{int}]	16337/6063 [0.0547]	31953/11915 [0.0694]	7955/7419 [0.0491]	35938/5009 [0.0484]
Completeness to θ (%)	99.7	99.6	67.9	100.0
Refinement method	Full-matrix least squares on F^2	Full-matrix least squares on F^2	Full-matrix least squares on F^2	Full-matrix least squares on F^2
Data/restraints/parameters	6008/0/349	11802/6/574	5009/0/460	4956/0/238
Goodness-of-fit on F^2	0.997	0.894	0.929	1.066
Final R indices [$I \ge 2\sigma(I)$][a]	R_1 = 0.0423, wR_2 = 0.0798	R_1 = 0.0592, wR_2 = 0.1379	R_1 = 0.0483, wR_2 = 0.0878	R_1 = 0.0329, wR_2 = 0.0862]
R indices (all data)[a]	R_1 = 0.0689, wR_2 = 0.0849	R_1 = 0.1042, wR_2 = 0.1518	R_1 = 0.0974, wR_2 = 0.0990	R_1 = 0.0388, wR_2 = 0.0884
Absorption correction	Multi-scan	Multi-scan	Multi-scan	Multi-scan
Largest diff. peak/hole (eÅ^{-3})	0.492 and -0.297	0.697 and -0.462	0.592 and -0.675	0.351 and -0.419

[a] $R_1 = \Sigma||F_o| - |F_c||/\Sigma|F_o|$; $wR_2 = [\Sigma[w(F_o^2 - F_c^2)^2]/\Sigma[w(F_o^2)^2]]^{1/2}$.
